# Socialization to professionalism in medical schools: a Canadian experience

**DOI:** 10.1186/s12909-015-0486-z

**Published:** 2015-11-17

**Authors:** Anna Byszewski, Jeewanjit S. Gill, Heather Lochnan

**Affiliations:** 1 Division of Geriatrics, Department of Medicine, University of Ottawa, Ottawa, Ontario Canada; 2 Department of Family Medicine, University of Western Ontario, London, Ontario Canada; 3 Division of Endocrinology and Metabolism, Department of Medicine, University of Ottawa, Ottawa, Ontario Canada

**Keywords:** Professionalism, Undergraduate, Curriculum

## Abstract

**Background:**

Accrediting bodies now recognize the importance of developing the professionalism competency, by setting standards that require medical schools to identify where professionalism is addressed and how it is evaluated within the formal curriculum.

The objective of this study was to compare how professionalism competency is formally addressed in the curricula of Canadian medical schools, and to better understand the Canadian approach to reporting and remediation of lapses.

**Methods:**

A literature review was performed and with the input of the AFMC(Association of Faculties of Medicine of Canada) Professionalism group, questionnaires were generated. An electronic survey was circulated to key leaders across the country at all the medical schools. In-depth telephone interviews were used to further explore themes, and a subsequent focus group was held to discuss challenges, particularly related to reporting and remediation.

**Results:**

The preponderance of formal professionalism teaching remains in the form of lectures and small group sessions in the preclinical years. Formal teaching declines significantly in the clerkship/clinical years. Evaluation is usually performed by a clinical supervisor, but OSCE, portfolio, and concern notes are increasingly used. Role modeling is heavily relied upon in clinical years, suggesting faculty training can help ensure clinical teachers recognize their influence on trainees. Formal remediation strategies are in place at most schools, and often involve essay writing, reflection exercises, or completion of learning modules about professionalism. Lack of clarity on what defines a lapse and fear of reprisal (for both trainees and faculty) limits reporting.

**Conclusions:**

This study provides an overview of how professional identity formation is supported in the Canadian context, guided by the standards set out by CanMEDS. Despite a rich literature that describes the definition, program design and evaluation methods for professionalism, in some areas of the curriculum there is still an opportunity to ensure programs embrace the suggested framework. Examples of teaching and evaluation methods, deficiencies in the clinical years of study (clerkship) and challenges in addressing lapses and organizational structure are identified. The results help identify the gaps that need to be addressed and some solutions that can be modeled at other academic institutions.

## Background

Instilling the principles of professionalism and garnering interest in assessment of the learning environment is currently a key focus of accrediting bodies and medical training programs. Institutions must be able to identify where professionalism is being formally addressed including when and how it is being evaluated. This is even more challenging when we consider that there may not be a universally agreed upon definition of the term “professionalism” [1]. Although there is still some controversy over the exact meaning of professionalism, most would agree on the need to assess behaviours with appreciation of the context [2]. A majority of institutions have also developed an operational framework of what constitutes professional behaviour [3]. In order to have an effective evaluation and remediation process, professionalism standards need to be explicit, instructional methods should inspire learners, and programs ought to ensure that students value professionalism assessment [4].

Canada introduced the revised CanMEDS framework in 2005, “Professional” being one of the 7 physician roles (these include: clinician, scholar, manager, communicator, collaborator, health advocate and professional) [5]. This framework is currently undergoing a revision for end of 2015, but at the time of the project Canadian schools used this version:

**Table:** 

The description for the “Professional” role is as outlined: *“Physicians have a unique societal role as professionals who are dedicated to the health and caring of others. Their work requires the mastery of a complex body of knowledge and skills, as well as the art of medicine. As such, the Professional Role is guided by codes of ethics and a commitment to clinical competence, the embracing of appropriate attitudes and behaviors, integrity, altruism, personal well-being, and to the promotion of the public good within their domain. These commitments form the basis of a social contract between a physician and society. Society, in return, grants physicians the privilege of profession-led regulation with the understanding that they are accountable to those served”.*	

Initially CanMEDS was developed and adopted in pursuit of excellence by the Royal College of Physicians and Surgeons of Canada, which sets standards for medical and surgical specialties. The CanMEDS framework is also now accepted by the College of Family Physicians in Canada (referred to as CanMEDS-FM) for setting educational standards. CanMEDS roles have also been adopted in many other jurisdictions and countries, or have a version that similarly incorporates professional standards. Accreditation of postgraduate training programs based on utilization of the CanMEDs roles has gone a long way to ensure these roles, including professionalism, are explicitly taught, that learning programs develop objective driven curricula based on these roles, and that programs evaluate (at both undergraduate and postgraduate levels) the professionalism competency. In addition, North American medical schools are accredited using the LCME standards. The standard M31-A was introduced specifically to ensure learners have an environment where the attributes of professionalism are taught explicitly and evaluated, along the pathway to development of their professional identity [6]. Canadian medical schools have now also introduced the CACMS (Committee on Accreditation of Canadian Medical Schools) criteria, which include student instruction on medical ethics and assessment of the learning environment, as the future accreditation standard for medical programs [7]. Medical schools across the world are also implementing formal curricula to address the topic of professionalism. One of the first surveys, which included 117 US medical schools, was conducted in 1998, and reported that only 27.8 % had a dedicated professionalism curriculum spanning all years and that 64 % had implemented explicit evaluation methods [8].

Cruess and Cruess argued that for medical schools to meet societal expectations of future doctors, professionalism must be taught explicitly and that the foundation must be supported by a cognitive foundation [9]. This entailed formalized teaching of the definitions and expectations of medical trainees as they become socialized to the profession. More recently these authors have also recognized the importance of professional identity formation (PIF), a process of becoming a “good physician”, and that the learning environment is of essential importance in supporting the development of this competency [10]. A recent systematic review, first of its kind, undertook the task to identify evidence based studies on how professionalism curricula should be delivered [11]. This study recognized that the greatest influence on behaviour is by clinicians that the students encounter, who then become their role models. One can stipulate that professionalism competency development requires a hybrid approach, both the explicit teaching and the role modeling in the learning environment. Both forces need to be supported by a strong framework in the medical curriculum [12].

In addition, a systematic review from the United Kingdom summarized evidence on methods for developing professionalism by identifying five essential themes for developing professionalism: curriculum design, student selection, teaching and learning methods, role modelling and assessment methods [13].

An example of an innovative curriculum is a Personal and Professional Development program (PDD) integrated in Australia through an entire medical program. The “PPD intensive” is focused on professionalism and patient safety, consisting of didactic, small group and interactive sessions, held over 2 days, followed by reflection on a patient case narrative [14].

Previous work by Papadakis et al. has also identified a positive correlation between disciplinary actions by state medical boards against practicing physicians with documented incidents of unprofessional behaviour during medical school [15].

Further work done to explore the behaviours that are most strongly linked to future disciplinary actions, report that these often include incidents of irresponsibility, poor initiative, lack of motivation and severely reduced capacity for self improvement and insight during their training [16]. As learners enter the medical school system they are eager to pursue objective driven didactic and experiential learning of the clinical base. Often professionalism may be seen as difficult to define, subjective, and of lesser importance or relevance to students, who do not imagine themselves embroiled in an unprofessional encounter. Students have used the word ‘cringe” to describe how they feel when confronted with this aspect of their programs, and that they are likely to feel resentment and lack of engagement in the process [17]. Not only must training programs ensure a formalized professionalism curriculum exists in order for programs to thrive and to gain learners’ approval, but teachers must also develop innovative instructional methods and support this with a cadre of teachers who are exemplary role models.

## Objectives of the project

The goal of this project was to compare and explore how professionalism is addressed among Canadian medical schools. The administrative structure of the professionalism program, methods of program delivery in pre-clerkship and clerkship, student evaluation processes, as well as lapse reporting and remediation were explored through an electronic survey, telephone interviews and focus groups. In addition, the project was used to identify resources used at different institutions that could be shared or to help sustain programs. This exploratory process of professionalism programs across the country can be used by Canadian medical schools to compare themselves to national averages, and further serves as a barometer for medical schools in other countries to use.

## Methods

This study had three components, with the aim to obtain as much information from key persons responsible for professionalism program administration at all of the 17 medical schools in Canada. Data acquisition utilized triangulation, including an electronic survey, telephone interviews and a focus group conducted in 2012–2013. The study received research ethics approval from The Ottawa Health Sciences Network REB.

- Survey: The survey was developed with the assistance of the members of the Association of Faculties of Medicine of Canada (AFMC) Group on Professionalism, with several drafts revised for consistency. The electronic survey was distributed via Survey Monkey (30 questions) to the person identified as most responsible for the professionalism curriculum at each of the 17 Canadian Medical schools. Several reminders were required to improve the response rate. Questions were a mixture of multiple choice and short answer format and explored current professionalism teaching methods, evaluation and remediation process.
- Telephone interview: In-depth telephone interviews were conducted to further explore themes obtained from the electronic survey, with a focus on the remediation process. A guide with questions to lead the telephone interview was developed.
- Focus group: As experts in the field, members of the AFMC (Association of Faculties of Medicine of Canada) Group on Professionalism were invited by email and an electronic flyer to join a focus group immediately following the presentation of the results of the survey and telephone interviews at the 2013 Annual AFMC Professionalism meeting. A focus group guide was used to explore further the themes presented, and explore specifically issues around teaching in clerkship, role modeling, lapse reporting and remediation. Ten members agreed and signed a consent form to participate. Probe questions were used by a group facilitator and the session was audio-taped. The recording was transcribed verbatim and results were collated with all identifiers removed.

## Results

### Survey

At the time of the project, 14 of the 17 (82 % response rate) Canadian Medical schools were able to respond to the electronic survey. Presentation of the results is based on the 14 schools that responded.

With respect to the program structure (Table 1), 9/14 (64 %) of the medical schools currently have a professionalism website and 50 % have a formal professionalism office. At some institutions the role of the Director of Professionalism is shared with a Leadership or a Gender and Equity position. Eleven (71 %) of the medical schools have some version of a “White Coat” ceremony at the start of medical school to formally introduce the students to professional identity. Further to this, 79 % of schools reported that some type of assessment of professionalism is used in their admission criteria. Formal staff training or faculty development around professionalism is conducted at 6/14 schools (43 %). Only 36 % of respondents noted they have formal recognition or awards for professionalism; again another mechanism that can be used to identify role models who can serve to exemplify professionalism to their colleagues.

**Table 1:** Percentage of Canadian Faculties of Medicine with various professionalism components

Professionalism Component	Yes	No
Professionalism Website	64 % (9/14)	36 % (5/14)
Professionalism Office	50 % (7/14)	50 % (7/14)
White Coat Ceremony	79 % (11/14)	21 % (3/14)
Formal Staff Training on Professionalism	43 % (6/14)	57 % (8/14)
Formal Orientation to Professionalism for Medical Students	86 % (12/14)	14 % (2/14)
Ability to Contact Professionalism Director via E-mail or Telephone	93 % (13/14)	7 % (1/14)
Professionalism as Part of Admission Process	79 % (11/14)	21 % (3/14)
Professionalism Awards or Recognition	36 % (5/14)	64 % (9/14)
Evaluation of Professionalism of Professors or Clinical Preceptors	79 % (11/14)	21 % (3/14)
Remediation for Unprofessionalism	93 % (13/14)	7 % (1/14)

**Fig. 1:**
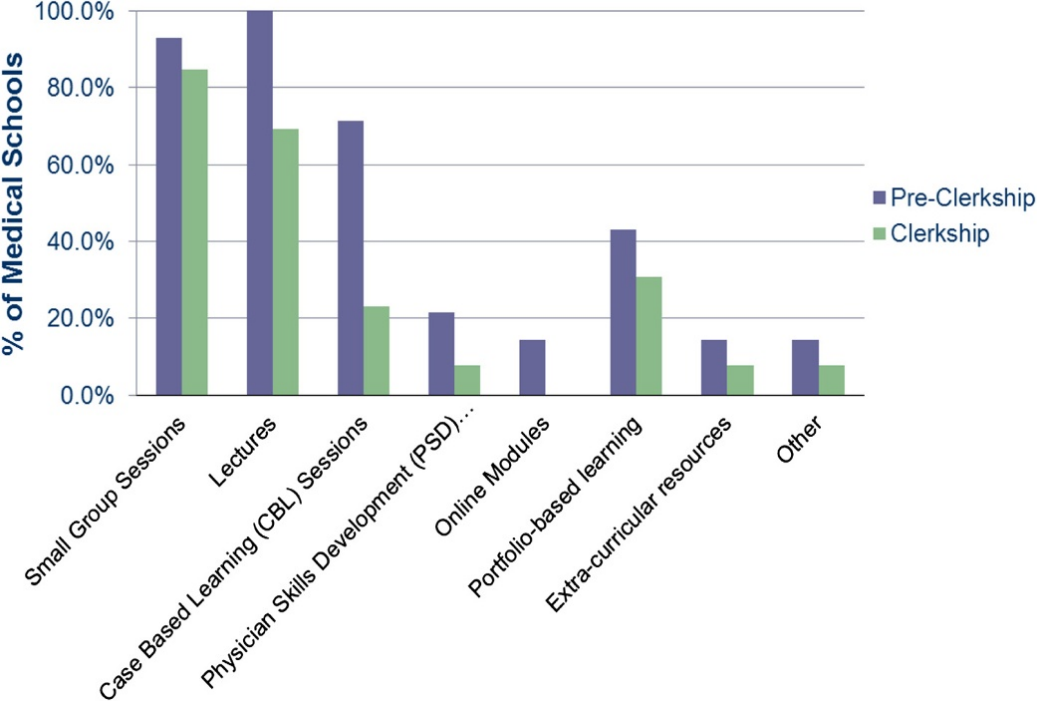
Comparison of teaching methods of professionalism pre-clerkship vs. clerkship)

In both pre-clerkship and clerkship, the foundation of teaching remains in the form of formal lectures and small group sessions (Fig. 1). Portfolios, consisting of reflective exercises documenting development of professionalism competency, are now used by 42.9 and 30.8 % respectively. Podcasts are being explored at some schools and might be considered supplementary to a formal curriculum.

**Fig. 2:**
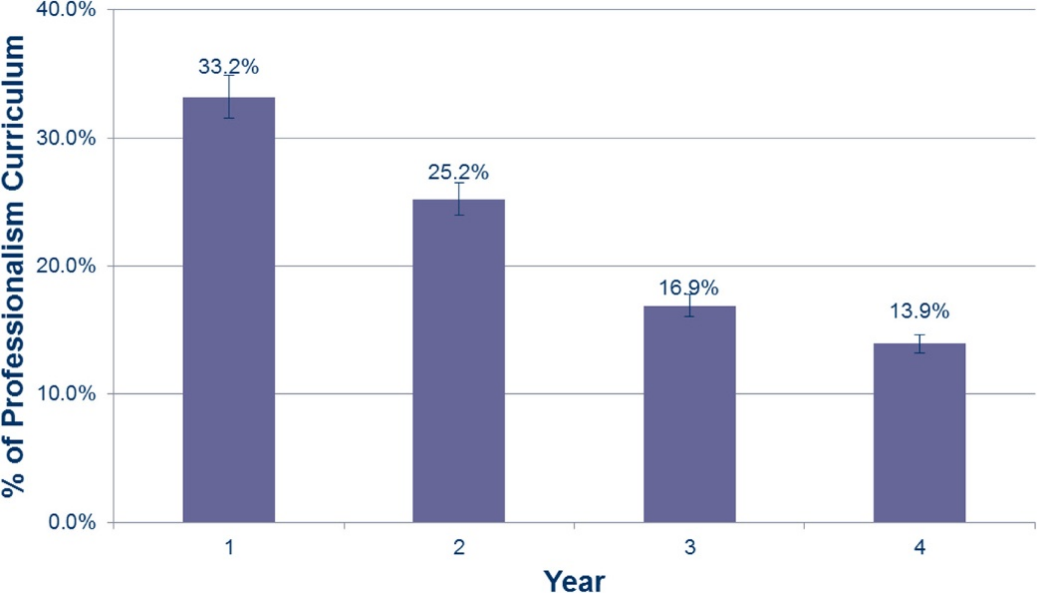
Comparison of relative amount of formal professionalism curriculum in each year of medical school

**Fig. 3:**
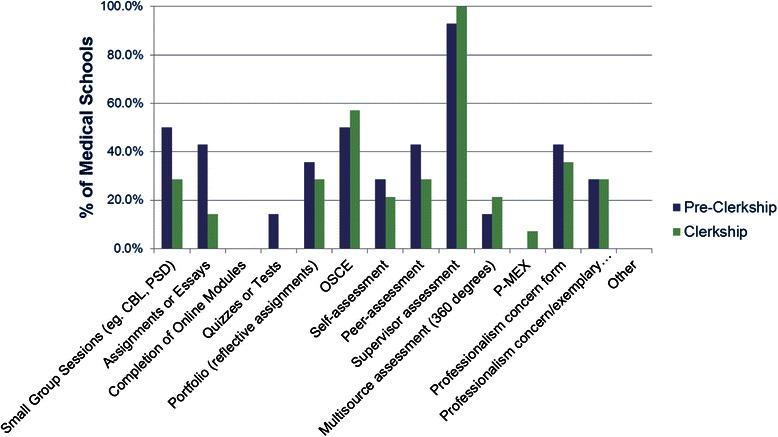
Comparison of evaluation methods of professionalism (pre-clerkship vs. clerkship)

The relative amount of formal professionalism curriculum (Fig. 2) decreases steadily from pre-clerkship to clerkship. Most of the formal teaching (33.2 %) is delivered in the first year, with only 13.9 % of the formal teaching occurring in the fourth year. Evaluation methods vary across the schools (Fig. 3), with periodic evaluation by supervisors during the pre-clerkship and clerkship being the most common method of evaluation. Other methods include OSCE and the option to complete concern notes in instances of lapses. Evaluation tools that are specific to professionalism, such as the PMEX [18] or multisource feedback, are being introduced at some centres. Celebrations of professionalism, such as awards or special recognition of professionalism, are now established at 5/14 (36 %) of the schools.

Remediation methods vary across the schools and include, at most centres, a meeting with the professionalism director or their representative. Remediation essays or exercises, and completion of modules, including reflection, are some of the remediation methods utilized. Seven (50 %) of the schools make use of a mental health assessment and probation mechanisms are in existence in 9/13 (64 %).

### Telephone interview

Five centres participated in the telephone interview, conducted by JG. The interview focused specifically on remediation. Those interviewed indicated that their institution adequately addresses professionalism issues and evaluation. Responsibility for providing input into clinical evaluation of a student’s professionalism in clerkship includes; clinical supervisors, residents, student peers, allied health, and in some cases patients. When unprofessional behavior is identified, it is generally a faculty member who has a discussion with the student.

Respondents indicated that remediation might not be necessary if it is a minor lapse and likely a one time event. Although it might not require formal remediation, the lapse should be documented, in case a pattern of lapses evolves. On the other hand, in some situations it may be futile to remediate in presence of an egregious lapse, lack of insight, lack of ability to reflect or in the case of repeated events, thus sanctions might need to be imposed, including dismissal from the program.

The most common types of professional lapses that were identified by the respondents included; confidentiality and privacy issues (hallway or sidewalk conversations), communication lapses (whether email or face-to-face) and issues around absences (not advising clinical supervisors or small group leaders). Other examples included improper attire and tardiness in handing in assignments.

Professional lapses identified as being most egregious included: actions that interfered with patient care, a breach of confidentiality related to patients’ health information, or criminal acts. Other examples included crossing boundaries with patients or co-workers, harassment, lying or falsifying records. The main barriers to reporting concerns about professionalism by students/residents/faculty included a fear of negative repercussions (Table 2).

**Table 2:** Telephone interviews: sample quotes on barriers to reporting

1. Fear of Negative repercussions:
*“[Residents are] worried about future job prospects and evaluation”*
*“For students, fear of reporting a faculty member or resident because they are being evaluated by them.”*
*“For staff/faculty… receiving a negative evaluation by the student, especially when faculty need to have valid positive evaluations when going for a promotion or if program is being evaluated by accreditation.”*
*“..reporting being perceived as subjective, but people are getting more comfortable…”*
2. Too much work involved
*“[The] amount of effort and work involved. I think people perceive it to be more work for themselves!”*
3. Futile
*“[Many people] feel nothing is going to be done anyways (laissez faire) – pointless. They are not going to invoke change anyway. Nobody is going to do anything anyway*

In most institutions 4/5 (80 %), the remediation process is not explicit or is done only on a case-by-case basis. It is important to emphasize that those less involved in teaching (such as preceptors in community or in more remote areas outside the academic centres) were felt to have more barriers in reporting and remediating, due to lack of knowledge of the process and policies. The organizational structure of Professionalism Offices also varied. The results of the telephone interview seem to indicate a lack of standardization across Faculties of Medicine as well as a lack of profile or status.

### Focus group

The focus group members consisted of 10 professionalism experts in Canada that attended the annual AFMC Professionalism Interest Group meeting and agreed to participate in the focus group. The focus group was led by a research assistant. Discussion ensued to address some of the major gaps in professionalism curricula identified in the surveys and telephone interviews: this being primarily clerkship teaching, and reporting and remediation of lapses.

1. Clerkship issues: the teaching of professionalism during clerkship on clinical rotations was felt to be difficult to capture. Although formal curriculum dedicated to the topic of professionalism is less evident in clerkship, this does not necessarily mean there is less teaching overall. Rather, it is a different, more of an experiential and informal form of learning for the trainee. The informal curriculum is part of the apprenticeship model with a strong need for role modeling. Although sometimes referred to as the hidden curriculum [19], it is more of a pseudo-hidden curriculum; if one looks closely professionalism issues are everywhere. The challenge thus falls on faculty to identify, and to bring to light, dilemmas or lapses to be used as learning opportunities. Even if there are negative role models, one can learn from these experiences if they are revealed, provided there is guidance (and not normalization of bad behavior). Professional identity formation occurs throughout the course of medical school, with a major shift in third year or early clerkship. Part of this process in later years of medical school involves struggling with complex issues encountered in a clinical context and ensuring there is a structure to support the learners in that process. Participants commented that portfolios or debriefing sessions can assist in this measure. There is a need to ensure this process is available to all of our learners, wherever they are in urban, rural or distributed sites.
2. Reporting and remediation: The group agreed there were significant differences across the country on how lapses are approached, with a general lack of standardization.

A consensus on a common approach was suggested and that the AFMC Professionalism Group could provide support through the dissemination of guidelines for remediation, which is a current work in progress.

As an example of the disparity across the country, some provincial licensing colleges in Canada require students to register as student members and require mandatory disclosure of a criminal record, whereas other provinces do not track this information on medical students. There is also variation on what is recorded on the Dean’s letters used as part of the application process for postgraduate training programs. One explanation may be that the institution does not want to jeopardize a student’s application by recording incidents of unprofessional behavior.

Another challenge that was discussed further in the focus group included the question of what is a major or minor lapse situation. Examples of minor lapses included being late for or absent from class, and major lapses could include a criminal act or stealing property from the hospital. Participants recognized the importance of reviewing the context of the situation and if acts were of a repetitive nature. Documentation of lapses was recognized as crucial to the process. Focus group members agreed to explore these issues in future to achieve consensus and to develop standards.

## Discussion

We have effectively described the Canadian landscape in regard to how Canadian Medical Schools formally address the topic of professionalism. This evaluative strategy incorporated a triangulated approach (survey, telephone interviews and focus group) to determine the professionalism curricula as seen by those that are most responsible for addressing professionalism at Canadian medical schools. Results reveal many of the challenges facing academic medical centers and thereby inform future direction at a national and local level. Further to this, other medical schools may be able to use this information when reviewing their own professionalism programs.

If we compare to a previous student survey from 2007 [20], clearly new teaching methods have now been developed and are adopted (portfolios, websites, podcasts, e-learning) but a lack of formal teaching in clerkship remains a challenge. Some factors that may contribute include students being placed in different clinical settings, no longer in one large classroom, which makes ensuring a consistent teaching or modeling of professionalism to the entire cohort is a challenge. The focus in clerkship shifts to the management of complex medical problems and may direct attention away from developing the non-clinician roles, such as professional. Yet this is the time that “critical events” are a fertile soil for professional identity formation, including introducing reflective writing [21]. Recent work on characteristics of the current cohort of medical students (Generation Me) also identifies them as having higher assertiveness, narcissistic traits, higher expectations and higher stress and anxiety than previous generations [22, 23]. These findings suggest that they may benefit from more structured learning but which is at the same time more interactive and experiential, including use of multimedia, online modules and portfolios.

Our results also indicate that most of the formal curriculum is being delivered in pre-clerkship. This is most likely due to the difficulty in delivering didactic sessions when learners are dispersed in clinical areas and no longer in classrooms during clerkship. However, as evidenced by the sentiments of the focus group, the professional identity formation is foremost at the time of clinical challenges, and leaves the “burden” for learning professionalism on role modeling. This is well supported by the recent systematic review that speaks of the importance of role modeling and clinician influence [11]. Novel methods are being also instituted, such as the longitudinal thread Professionalism Behavior (PB) designed in The Netherlands at the VUmc School of Medical Sciences in Amsterdam [24]. PB is a longitudinal thread embedded in the medical curriculum with well trained faculty, where teachers are given instruction or reporting unprofessional behaviors and are involved in remediation. Teachers were less reluctant to award an unsatisfactory professional behavior judgment, with remediation viewed as being helpful to the student. More detailed evaluations of the learning environment are also being introduced [25, 26] that will shed more light on the current status as well as hopefully engage clinicians in the role modeling process.

Further studies are also needed to determine whether the less formalized curriculum in clerkship might explain concerns that professionalism and empathy diminish as training proceeds and whether a formal curriculum could prevent this trend. Hojat et al., described how “Devil is in the Third Year” [27]. In their paper they describe how medical students’ performance on empathy rating scales significantly diminished in the third year, just as their involvement with patients ramps up. One explanation put forth was a form of “social amnesia” as described by Hafferty [28].

Procedures for evaluating student professionalism are in place in most programs in Canada, but largely depend on evaluation by supervisors in both pre-clerkship and clerkship. Standards may not be clearly defined with a paucity of actual observable behaviors evaluated [29]. Millar’s pyramid provides a useful framework for developing a connection between level of competence and assessment methods [30]. Evaluation methods such as PMEX (Professionalism Mini exercise), OSCE (Objective Structured Exam) and MSF (Multisource feedback) are being implemented in our programs where a higher level competence can be objectively evaluated. The CanMEDS 2005framework in operation at the time was not entirely explicit in recommending specific strategies for program delivery but it provides some assessment tool suggestions for the professional role, including direct observation, multisource feedback, and portfolios. Our results (Figs. 1 and 3), indicate that most schools use direct observation, but just over 40 % use portfolios and 23 % use multisource feedback [31].

We also identified the major challenge of reporting lapses and remediation. The survey established that several strategies for remediation are emerging. Those interviewed spoke to the difficult task of reporting, with fear of reprisal, both on the part of trainees and faculty. It is also often unclear what is the exact process and outcome, with added apprehension of the time and effort required to pursue the reporting process. This is consistent with recent work done by the authors in this area, as faculty are often loathe to comment on or report deficiencies in professional behavior [32].

The focus group further identified the ambiguity and lack of standardization that occurs when trying to identify what is a minor or major lapse. The participants emphasized that context, as conceptualized by Ginsburg, is vital in categorizing and understanding lapses [2].

Future directions will require the incorporation of innovative methods of program delivery that could be designed to engage students by using technology. In particular, more formalized curriculum should be inserted into clerkship where currently there is very little time dedicated to professionalism and where role modeling is so important and diminished professionalism is at risk. A recent study of postgraduate directors in Canada on key elements of the professional role identified attitudinal attributes of honesty and integrity as being most important, with morality and codes of behavior being the next selected essential component [33]. Yet current formal postgraduate teaching emphasizes biomedical ethics and legal frameworks, which were felt to be of much less importance. This gap draws further attention to the need of increasing faculty awareness of their influence as role models and for the required support for training of clinical teachers. Faculty development addressing professionalism should be encouraged, as well as recognition strategies, and most importantly the effective management of negative role models must be given high priority. Warren et al. report that program directors were not optimistic in the overall success of their own faculty development initiatives, due to lack of faculty engagement and the fact that many faculty members have limited preparation for teaching, evaluating, and remediating professionalism [33].

We are unlikely to eliminate unprofessional behavior completely so a reporting and remediation mechanism must be in place. Identifying explicitly the pathways to reporting and defining the lapses and remediation methods with clear lines of communication will help to standardize the process. Finally, continuous surveillance and formal evaluation of the learning environment may direct the establishment of exemplary role models to guide development of professional identity of the physicians of tomorrow.

## Limitations

Our project had some limitations. We were able to survey 14 of the 17 medical schools, as the electronic survey was conducted over the summer holiday period, while JG (medical student at the time) was available to conduct the project delivery. However, the 82 % response rate probably reflects the current status across the country. The information pertains to the status in Canadian medical schools and may not apply to other centers. However, we know that increasingly accrediting bodies are placing a stringent requirement for medical schools to address professionalism. We assume that our results are likely widely generalizable to medical schools internationally which use a pre-clerkship/clerkship model of training.

## Conclusions

The medical education literature has developed greatly over the last three decades in the area of professionalism, including the challenges of the definition, on how to inspire students on the topic, and different methods of delivery of teaching and evaluation. We used the CanMEDS format as a standard for program delivery in our review of programs in Canada, in addition to the literature review. Although with this project we have seen some further development of innovative methods over the 5 years as compared to a previous study (eportfolios, reflective writing exercises, multisource feedback, etc.), there remains room to improve on the delivery of the curriculum, particularly in clerkship and around reporting of lapses and remediation. If we are to support the professional identity formation of the physicians of tomorrow, we owe it to our learners to meet this challenge.

## Practice points

1. Medical schools in Canada are adopting innovative strategies to teach and evaluate professionalism which include portfolios that promote reflection, small group case based sessions, OSCE’s and webinars.
2. The delivery of formal professionalism curriculum content continues to be more prominent in pre-clerkship than in clerkship (clinical years) when role modeling becomes the predominant means of developing students’ professional identity. Faculty development may help ensure teachers understand their influence as role models.
3. Reporting and remediation processes vary across medical schools. Canadian experts agree that standards are needed and are working to develop national recommendations.
4. Most medical schools recognize the need for leadership and resources to adequately address professionalism and most medical schools in Canada have created an office dedicated to professionalism issues.
